# MRI Imaging of Uterine Incarceration: From Diagnosis to Posttreatment Follow‐Up

**DOI:** 10.1155/crog/4993782

**Published:** 2026-10-06

**Authors:** Natália Henz Concatto, Ariane Giovanaz, Meiri Andreia Maria da Silva, Rubia Vanceta, Camila Braga Visconti, Adriana Prato Schmidt, Carolina Paz Mohamad Isa, Marcela Godoy Dias, Caroline Lorenzoni Almeida Ghezzi, Janete Vettorazzi, Cristiano Caetano Salazar

**Affiliations:** ^1^ Postgraduate Program in Health Sciences: Gynecology and Obstetrics Federal University of Rio Grande do Sul (UFRGS) Porto Alegre Rio Grande do Sul Brazil ufrgs.br; ^2^ Radiology Department Hospital Moinhos de Vento Porto Alegre Rio Grande do Sul Brazil hospitalmoinhos.org.br; ^3^ Service of Obstetrics and Gynecology Hospital Moinhos de Vento Porto Alegre Rio Grande do Sul Brazil hospitalmoinhos.org.br; ^4^ Service of Obstetrics and Gynecology Hospital de Clínicas de Porto Alegre Porto Alegre Rio Grande do Sul Brazil ufrgs.br; ^5^ Radiology Department Hospital de Clínicas de Porto Alegre Porto Alegre Rio Grande do Sul Brazil ufrgs.br

**Keywords:** diagnostic imaging, incarcerated gravid uterus, magnetic resonance imaging, pregnancy, urinary retention, uterine incarceration

## Abstract

**Introduction:**

Uterine incarceration is a rare and frequently underrecognized obstetric complication caused by persistent retroversion of the gravid uterus. Because clinical manifestations are often nonspecific, diagnosis may be delayed, increasing the risk of maternal and fetal complications. Imaging, particularly magnetic resonance imaging (MRI), may be essential when ultrasound findings are inconclusive.

**Case Presentation:**

A 37‐year‐old multigravida with a history of recurrent pregnancy loss presented at 16 weeks′ gestation with acute urinary retention. Clinical examination raised suspicion for uterine incarceration. MRI confirmed the diagnosis by demonstrating a retroverted gravid uterus impacted within the posterior pelvis, associated with elongation and anterior displacement of the cervix and compression of the lower urinary tract. Prompt bladder decompression and successful uterine repositioning were performed, with subsequent resolution of symptoms and favorable maternal‐fetal evolution.

**Discussion:**

This case highlights the importance of correlating clinical and imaging findings in pregnant patients presenting with acute urinary symptoms. MRI was decisive in clarifying pelvic anatomy and establishing the diagnosis when sonographic evaluation was limited. Early recognition of uterine incarceration enables timely intervention and may reduce the risk of obstetric and urological complications, contributing to improved maternal‐fetal monitoring and management.

**Learning Points**

• Acute urinary retention in the second trimester may represent the first manifestation of uterine incarceration.
• MRI clarified the altered pelvic anatomy by demonstrating a retroverted incarcerated gravid uterus with anterior displacement of the bladder and elongated cervix.
• Recognition of characteristic MRI findings may facilitate earlier diagnosis when ultrasound assessment is inconclusive.
• Radiologists must be aware of potential imaging pitfalls and clinical mimics, including placenta previa, Müllerian anomalies, and pelvic masses.

## 1. Introduction

Incarcerated gravid uterus, also referred to as trapped uterus, is a rare but potentially severe obstetric complication that occurs when a retroverted gravid uterus becomes impacted within the posterior pelvis and fails to ascend into the abdominal cavity during pregnancy progression. Although uterine retroversion is observed in up to 15% of pregnancies during the first trimester, spontaneous correction usually occurs between 14 and 16 weeks′ gestation. Persistent incarceration may lead to maternal and fetal complications, including urinary obstruction, uterine rupture, obstructed labor, and fetal growth restriction [1, 2].

Clinical diagnosis is often challenging because symptoms are variable and nonspecific, ranging from pelvic pain and urinary complaints to asymptomatic presentations. Acute urinary retention is considered one of the most characteristic manifestations and may represent the first clinical sign of incarceration. Ultrasound is typically the initial imaging modality; however, distorted pelvic anatomy and difficulty identifying the cervix and uterine position may limit sonographic evaluation. In these situations, magnetic resonance imaging (MRI) provides superior anatomical characterization and may confirm the diagnosis by clearly demonstrating the relationship between the gravid uterus, cervix, bladder, and surrounding pelvic structures [3].

In a normally positioned gravid uterus beyond the first trimester, the uterine fundus ascends above the sacral promontory, the cervix remains in its expected posterior position and maintains an approximately perpendicular orientation to the vaginal axis, and the urinary bladder retains its usual anterior pelvic position without marked superior displacement or extrinsic compression. In contrast, MRI findings suggestive of uterine incarceration include persistent uterine retroversion or retroflexion, with the fundus trapped deep within the posterior cul‐de‐sac below the sacral promontory; elongation, thinning, and anterosuperior displacement of the cervix, often oriented parallel to the vaginal axis; anterior stretching of the lower uterine segment; and anterior or superior displacement and distortion of the urinary bladder, which may extend above the pubic symphysis [4–6].

This report describes a case of uterine incarceration presenting with acute urinary retention at 16 weeks′ gestation in which MRI was essential for diagnosis and therapeutic planning after inconclusive ultrasound assessment. By emphasizing the imaging findings associated with urinary obstruction, this case contributes to the recognition of atypical presentations of uterine incarceration and highlights the role of MRI in guiding timely management.

## 2. Case Presentation

A 37‐year‐old gravida 6 patient with five previous pregnancy losses presented during a spontaneously conceived pregnancy. Her obstetric history included one complete miscarriage at 10 weeks, one miscarriage of a twin pregnancy at 7 weeks, and three biochemical pregnancies. The investigation for recurrent pregnancy loss was unremarkable, with no evidence of Müllerian anomalies or other identifiable pelvic abnormalities. She had no history of pelvic surgery, endometriosis, or uterine fibroids. During the first trimester, she received aspirin, progesterone, and vitamin supplementation. Gestational diabetes was diagnosed and managed with dietary measures.

At 12 weeks′ gestation, routine vaginal examination showed that the uterus was positioned low in the pelvis. At 16 weeks, the patient developed acute urinary retention requiring bladder catheterization. Initial differential diagnoses included urinary tract obstruction, neurogenic bladder dysfunction, and pelvic mass effect. Transabdominal ultrasound demonstrated marked bladder distension and abnormal uterine positioning, raising suspicion of uterine incarceration; however, the uterine position and cervical anatomy could not be adequately characterized. Because the clinical and sonographic findings were insufficient to establish a definitive diagnosis, pelvic MRI was performed and demonstrated a retroverted gravid uterus impacted within the sacral concavity, with elongation and anterior displacement of the cervix, confirming uterine incarceration (Figure 1).

**Figure 1:**
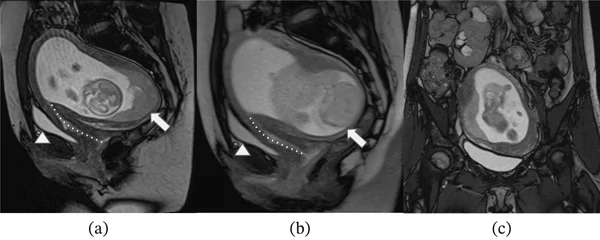
MRI images of uterine incarceration at 16 weeks of gestation. (a,b) Midline sagittal True FISP images demonstrate a retroverted gravid uterus with the fundus (white arrows) deeply impacted within the sacral concavity, lodged below the sacral promontory between the promontory and the pubic symphysis (arrows in a,b). The lower uterine segment is anteriorly stretched. The cervix appears elongated (dashed line in a,b), thinned, and cranially displaced, oriented parallel to the vaginal axis rather than in its normal perpendicular configuration. Note the marked anterior and superior displacement of the urinary bladder, with distortion of its contour due to external compression by the entrapped uterus. The bladder dome is elevated above the pubic symphysis (arrowheads in a,b), a characteristic finding that is often considered pathognomonic. (c) Coronal T2 True FISP demonstrating compression of the urinary bladder by the incarcerated uterus.

Initial conservative management with bladder decompression and positional maneuvers was unsuccessful. Manual uterine repositioning was subsequently performed under general anesthesia with the patient in lithotomy position after bladder emptying. Gentle upward pressure was applied digitally through the posterior vaginal fornix to mobilize the uterine fundus out of the posterior pelvis, resulting in immediate symptom resolution. A Gellhorn pessary was placed to maintain uterine position (Figure 2). The pessary was removed at 23 weeks after uterine ascent beyond the pelvis.

**Figure 2:**
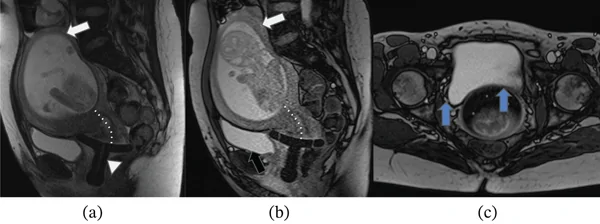
Control MRI images following manual repositioning in the same patient. (a,b) Sagittal T2 True FISP images demonstrating restoration of uterine anteversion, with the fundus relocated above the sacral promontory (arrows in a,b). The cervix is located posteriorly, oriented perpendicularly to the vagina, with longitudinal extension within normal limits (dashed lines in a,b). A vaginal Gellhorn pessary was placed to maintain uterine position (arrowhead in a). The urinary bladder shows a normal shape and usual position (blue arrows in b,c). (c) Axial T2 True FISP demonstrating the bladder with its typical shape, without signs of extrinsic compression.

The pregnancy subsequently progressed without major complications until 29 weeks, when reduced fetal growth velocity was observed, with estimated fetal weight at the 12th percentile and abdominal circumference at the 8th percentile. Although these findings did not fulfill formal criteria for fetal growth restriction, closer surveillance was initiated. Serial ultrasound examinations and Doppler studies remained within normal limits throughout follow‐up, allowing continuation of pregnancy until an uncomplicated elective cesarean section at 38 weeks. Postpartum follow‐up was uneventful, and the patient expressed satisfaction with the diagnostic and therapeutic management.

## 3. Discussion

Uterine incarceration is an uncommon obstetric complication, and its diagnosis can be particularly challenging because of its often subtle and nonspecific clinical presentation, especially in early pregnancy. Symptoms may include dysuria, pelvic discomfort, constipation, vaginal bleeding, and back pain. Urinary retention is considered a key clinical finding and should prompt further investigation [2, 3]. On physical examination, findings may include a palpable mass in the cul‐de‐sac and a markedly anterior and superiorly displaced cervix, which may be difficult or impossible to reach on vaginal examination [2, 3].

Several risk factors have been associated with uterine incarceration, including posterior uterine fibroids, endometriosis, pelvic adhesions, congenital uterine anomalies, a deep sacral concavity with a prominent sacral promontory, previous episodes of uterine incarceration, and abnormal placental implantation [2, 4]. However, up to 50% of cases occur in patients without identifiable predisposing factors at presentation [2, 4]. Similarly, no clear anatomical or pelvic predisposing condition was identified in our patient. Although causality cannot be established, progesterone therapy may have contributed to persistence of uterine retroversion or altered pelvic support dynamics.

Ultrasound is typically the first‐line imaging modality during pregnancy. In the first trimester and early second trimester, transvaginal and transabdominal approaches may be complementary in assessing uterine orientation, cervical position, and the relationship between the uterus and urinary bladder. Because uterine retroversion commonly resolves as the enlarging uterus ascends from the pelvis between 14 and 16 weeks′ gestation, persistence beyond this period, particularly in patients with urinary or pelvic symptoms, should prompt targeted evaluation for uterine incarceration. In later gestation, however, the enlarged uterus and distorted pelvic anatomy may make the cervix and the normal uterocervical relationship difficult to identify. In this setting, transabdominal ultrasound may provide a broader assessment of the cervix, internal os, vagina, uterus, and bladder [3–7].

When the diagnosis remains uncertain after ultrasound, MRI without gadolinium can play a pivotal role in confirming uterine incarceration and providing detailed anatomical assessment. MRI may be performed in any trimester when clinically indicated and should not be postponed solely because of gestational age. It may be particularly valuable during the late second and third trimesters, when sonographic assessment becomes more limited and detailed evaluation of uterine orientation, cervical elongation, bladder displacement, placental location, and associated complications may be required for therapeutic or delivery planning [4–8]. Its multiplanar capability, wide field of view, and excellent soft‐tissue contrast allow accurate evaluation of distorted pelvic anatomy and delineation of adjacent organs [1, 2, 5].

On MRI, uterine incarceration is typically characterized by persistent retroversion, with the fundus deeply positioned within the posterior pelvis below the sacral promontory (Figure 3). The cervix is usually elongated and thinned and may appear as a T2‐hypointense linear structure oriented parallel to the vaginal axis rather than in its expected perpendicular orientation [2, 6]. Another characteristic finding is distortion of the urinary bladder due to extrinsic compression and anterosuperior displacement by the gravid uterus [2, 5, 6]. In our patient, MRI was particularly valuable for demonstrating the marked distortion of the vesicouterine anatomy, correlating these findings with the acute urinary retention, and excluding alternative pelvic conditions that could mimic incarceration.

**Figure 3:**
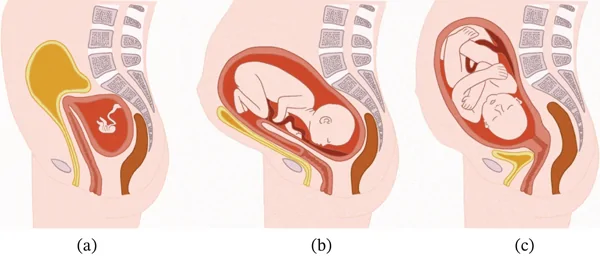
Schematic drawing of uterine incarceration created by the authors for educational purposes. (a) Uterine incarceration in the late first trimester. The uterine fundus is trapped in the posterior cul‐de‐sac and the lower uterine segment is wedged beneath the sacral promontory. The cervix is displaced anteriorly, lying behind the pubic symphysis, and the bladder is markedly distended and displaced superiorly. (b) Persistent uterine incarceration in the third trimester. The fundus remains the most posterior and inferior portion of the uterus, lodged between the pubic symphysis and the sacral promontory. The cervix is elongated and anteriorly displaced. Although the fetus appears to be in cephalic presentation, it is actually in complete breech. (c) Normal pregnancy with cephalic presentation. The uterus is anteverted, with the fundus located superiorly and the cervix in its expected posterior position. Illustration by Natália Henz Concatto, created using Procreate (Savage Interactive, Hobart, Australia). All authors have reviewed and verified the scientific content and accuracy of the illustration.

Several imaging pitfalls and clinical mimics may simulate uterine incarceration, potentially leading to delayed diagnosis or inappropriate management. Placenta previa may be falsely suggested on ultrasound because of the abnormal orientation of the incarcerated uterus. Other differential diagnoses include abdominal pregnancy, cervical or interstitial ectopic pregnancy, Müllerian anomalies, uterine torsion, and pelvic masses such as degenerating fibroids or large adnexal lesions [2, 5, 6]. In most cases, these entities can be reliably differentiated through careful evaluation of pelvic anatomy with ultrasound and, particularly, MRI.

Most obstetricians aim to restore the uterus to its normal anatomical position as soon as possible after diagnosis, although management strategies vary according to gestational age [3]. In early pregnancy, conservative measures and manual repositioning may be attempted, including gentle traction on the cervix combined with rectal pressure on the uterine fundus to release it from beneath the sacral promontory [3, 9, 10]. Epidural or general anesthesia may facilitate reduction by promoting pelvic muscle relaxation, and pessary placement has been described to maintain uterine position after successful repositioning [3]. Surgical intervention, usually by laparoscopy or laparotomy, is reserved for refractory cases [3, 9, 10]. In our patient, early diagnosis and prompt management were likely important factors contributing to successful uterine repositioning and avoidance of surgical intervention or severe maternal complications.

Delayed diagnosis may lead to significant maternal and fetal morbidity, including persistent urinary obstruction, renal failure, uterine sacculation or rupture, obstructed labor, fetal growth restriction, preterm delivery, and intrauterine fetal demise [2, 5, 6]. In the second and third trimesters, persistent malposition of the cervix may impair cervical dilation and increase the risk of difficult delivery and iatrogenic injury during cesarean section, particularly involving the bladder or cervix [5, 6]. In our patient, despite favorable maternal evolution after successful manual repositioning, fetal growth surveillance remained necessary throughout pregnancy. Early recognition of imaging findings is therefore essential not only to guide management, but also to optimize obstetric planning and reduce maternal‐fetal morbidity.

## 4. Conclusion

This case underscores the value of MRI as a problem‐solving tool when sonographic assessment of suspected uterine incarceration is inconclusive. By providing precise anatomical information before intervention and documenting restoration of normal pelvic relationships after treatment, MRI can support therapeutic planning, multidisciplinary decision‐making, and posttreatment follow‐up. Greater familiarity with its imaging appearance may facilitate timely recognition of this uncommon condition and help prevent avoidable maternal and fetal complications.

## Author Contributions

**Natália Henz Concatto:** conceptualization, writing – original draft, writing – review and editing, Supervision. **Ariane Giovanaz:** writing – original draft, writing – review and editing. **Meiri Andreia Maria da Silva:** writing – original draft, writing – review and editing. **Camila Braga Visconti:** writing – review and editing. **Rubia Vanceta:** writing – review & editing. **Adriana Prato Schmidt:** clinical data acquisition, writing – review and editing. **Carolina Paz Mohamad Isa:** clinical data acquisition, writing – review and editing. **Marcela Godoy Dias:** writing – review and editing, supervision. **Caroline Lorenzoni Almeida Ghezzi:** writing – review and editing, supervision. **Janete Vettorazzi:** conceptualization, writing – review and editing, supervision. **Cristiano Caetano Salazar:** conceptualization, writing – review and editing, supervision.

## Funding

No funding was received for this manuscript.

## Disclosure

Natália Henz Concatto had full access to all of the data in this study and takes complete responsibility for the integrity of the data and the accuracy of the data analysis. Natália Henz Concatto affirms that this manuscript is an honest, accurate, and transparent account of the study being reported; that no important aspects of the study have been omitted; and that any discrepancies from the study as planned (and, if relevant, registered) have been explained. All authors have read and approved the final version of the manuscript.

## Ethics Statement

This study was conducted in accordance with the ethical standards of the institutional research committee and with the 1964 Helsinki Declaration and its later amendments. Formal ethics committee approval was not required for this case report in accordance with local institutional guidelines. Patient confidentiality was maintained throughout, and no identifying information is included in this manuscript.

## Consent

Written informed consent was obtained from the patient for the publication of this case report and accompanying images. A copy of the written consent is available for review by the Editor‐in‐Chief of this journal upon request.

## Conflicts of Interest

The authors declare no conflicts of interest.

## Data Availability

The data that support the findings of this study are available on request from the corresponding author. The data are not publicly available due to privacy or ethical restrictions.
